# 96. Does a structured approach in the form of a rheumatology virtual biologics clinic improve patient safety and efficiency?

**DOI:** 10.1093/rap/rky032.004

**Published:** 2018-09-20

**Authors:** Muhamad Jasim, Gurmit Smith, Sharon Petford, Lisa Jones, Holly John

**Affiliations:** Rheumatology, Dudley NHS Trust, Dudley, UNITED KINGDOM

**Introduction:** The use of biologic therapies in the treatment of inflammatory arthropathies have increased significantly in recent years as have the variety of biologics available for us to use. Many biologics, particularly anti-TNF antibody therapies, have been associated with an increased risk of latent TB reactivation. Therefore, risk assessment of patients is required as recommended by both the British Society for Rheumatology and British Thoracic Society (BTS) guidelines. However, currently, at a time when biologic therapies are more widely used, we are dealing with increased clinical pressures as well as a greater social mix and diversity in our patient populations that increase the challenge of identifying who is indeed a high risk patient for latent TB. This sub-optimal risk stratification was confirmed on a regional audit. It is therefore unsurprising, that virtual biologics clinics (VBC) have gained in acceptance and popularity. These VBC should in theory help to standardise and streamline the process from initial referral to commencement of the biologic including all the necessary screening required. The case below highlights a patient who was screened and commenced her biologic treatment prior to the initiation of a VBC at Russell’s Hall Hospital Rheumatology Department, Dudley. We then highlight the changes implemented with the initiation of our local VBC clinic. We later discuss results that highlight the impact the VBC seems to have made to enhance patient safety and efficiency.

**Case description:** In 2014, a 68 year old lady was urgently referred to the nurse specialist’s biologics clinic for initiation of biologic treatment for her poorly controlled rheumatoid arthritis despite the use of triple DMARD therapy with methotrexate, sulfasalazine and hydroxychloroquine. The patient was not identified by either the doctor referring the patient or by the nurse in clinic to have risks for TB. As per the BTS guidelines she underwent a chest x-ray which was normal (although not formally reported). Soon after, the patient was commenced on adalimumab in September 2014. However, a few months later in March 2015 the patient was seen in clinic where she reported a dry cough and night sweats. Further questioning revealed that the patient was born in El Salvador and travelled to Cuba as well as the Philippines regularly. The patient was referred to our TB specialist for further assessment and a T-spot test was positive. The patient was consequently commenced on chemoprophylaxis. The question is however, could this all have been pre-empted? Studies have found that the majority of cases of TB occur within the first 12 months of treatment with an anti-TNF agent suggesting that reactivation of latent TB is the main factor predisposing to TB. This case illustrated the clinical variability and inconsistency in the extent of risk assessment and highlights that existing guidelines can miss cases of latent TB. In fact, a study in 2013 found use of just the BTS guidance for screening immunosuppressed patients leads to missed cases of latent TB. In November 2016 Russell’s Hall Hospital implemented its own version of a VBC. In it a doctor, nurse and secretary work to confirm the suitability of the drug for each patient and review their eligibility and any contraindications. They would also review and chase all screening tests which were made uniform for all patients due to commence a biologic, this included a T-spot and ensuring a formal chest x-ray report was obtained. Finally, the necessary Bluteq forms would be completed and follow up appointments booked. Any patients found to be high risk would have an urgent referral to the chest physicians, with an agreed pathway set up. We have since managed to assess and analyse the impact of this VBC on our practice. We performed a retrospective study using a combination of rheumatology database, clinic letters as well as the hospital intranet. We compared all patients who were processed through the VBC clinic between November 2016 to September 2017 (N = 49) against 48 randomly selected patients selected from our biologic repeat prescription list. These 48 patients had been commenced on biologics between April 2013 and July 2016 (before the VBC was implemented). Anyone with insufficient information available on the database, in clinic letters or on the hospital intranet was excluded as were any patients swapping biologics. Demographic results for pre-VBC group included an average age 48 (range 24-83) vs VBC average age 52 (range 23-78). As expected the majority of both cohorts of patients had a diagnosis of rheumatoid arthritis. Of our 48 pre-VBC cohort, 18 underwent a T-spot test, with one positive results (6%). However, our 68 year old lady described earlier was one of the 30 patients who did not undergo a prior T-spot test. In contrast, 100% of the VBC cohort underwent a T-spot test with six patients (12%) having a positive result. Of our pre-VBC cohort 70% had their chest x-ray formally reported. In comparison 100% of the VBC patient group had a formal x-ray report. When comparing the cohort's efficiency in the time between initial referral to the commencement of the biologic we found the mean duration in our pre-VBC group was 143 days (range 22-376 days) vs 99 days in the VBC group (range 28-322 days). The median in the pre-VBC cohort was 127 days vs 82 days in the VBC cohort.

**Discussion:** Our clinical case described earlier highlighted the challenge frequently encountered in the screening and the risk assessment of patients referred for biologic initiation. The hope at Russell’s Hall Hospital was that the initiation of a virtual biologics clinic in the rheumatology department would help make the process safer and more efficient. The results described above underlines the impact the VBC has made. Overall, the median length of time from initial referral to biologic commencement has improved by 35%. Clearly several patients took extended periods of time. On further investigation reason often varied but included; the patient not attending appointments, positive hepatitis screen, requiring respiratory reviews due to TB risk and complications caused due to TB chemoprophylaxis. On further analysis of the six VBC cases who had a positive T-spot result we found each had a different risk factor: one was an ex-nurse, another was Asian, while one patient was Afro-Caribbean, one patient was a retired GP receptionist, another had a friend who had TB six years earlier. Finally, one patient had no risk factors at all. Therefore, one could argue how many of these would have been screened and identified to have latent TB prior to the VBC? Although, one could argue that the use of across the board uniform screening protocols add extra expense this would pale into comparison against the cost of a single patient requiring admission for TB especially if it could have been pre-empted and prevented. In our sample group an extra £1,470 total was spent performing tests on every VBC patient.

**Key Learning Points**: Implementation of a virtual biologics clinic in Russell’s Hall Hospital has been found to have resulted i: better efficiency - improvement in average time from initial referral to commencement of biologic; safer - proved by finding non high risk latent TB cases and that all chest x-rays are now reported; standardisation of protocols = improved consistency. However, complex patients are always going to be complex and will require lengthy work-up prior to commencement of biologic regardless of a more structured framework.

**Disclosure: M. Jasim:** None. **G. Smith:** None. **S. Petford:** None. **L. Jones:** None. **H. John:** None.

